# Beneficial Role of Mg^2+^ in Prevention and Treatment of Hypertension

**DOI:** 10.1155/2018/9013721

**Published:** 2018-06-11

**Authors:** Andrea M. P. Romani

**Affiliations:** Case Western Reserve University, School of Medicine, Department Physiology and Biophysics, 10900 Euclid Avenue, Cleveland, OH 44106-4970, USA

## Abstract

Hypertension constitutes one of the most widespread pathological conditions in developed and developing countries. Currently, more than 1 billion people worldwide are affected by the condition, either as frank hypertension or as prehypertension, raising the risk for major long-term complications and life-threatening pathologies. The costs in terms of health care services, medications for the treatment of hypertension and its complications, and associated loss in productivity represent a major economic burden for the various countries. The necessity of developing treatments that are economically more sustainable and with better compliance has been increasing alongside the incidence of the pathology. Along these lines, attention has been paid to the implementation of affordable but nutritious diets that deliver appropriate levels of macro- and micronutrients as integral part of the diets themselves or as supplements. In particular, experimental and clinical evidence suggests that an appropriate intake of dietary magnesium can be beneficial in controlling blood pressure. Additional advantages of a more diffuse therapeutic and/or preventive utilization of magnesium supplements are the virtual absence of side-effects and their affordable costs. The present review will attempt to frame our knowledge of how magnesium exerts its beneficial effects on blood pressure maintenance, which may lead to the development of more effective treatments of hypertension and its main complications.

## 1. Introduction

Hypertension represents one of the most common human pathological conditions diagnosed. According to data published in 2013 by the World Health Organization, approximately 1.2 billion people worldwide are affected by the pathology [1], irrespective of developed or developing countries, ethnicity, gender, and age. Consistent with this trend, nearly one-third (>29% or 75 millions) of the American adult population is affected by hypertension, defined as high blood pressure [2], and another third presents with prehypertensive state [2], whereby blood pressure values are higher than normal but not yet in the high blood pressure range. More worrisome, only half of the individual with frank hypertension have their condition under medical control [2], raising the risk for the occurrence of life-threatening cardiovascular complications including stroke, myocardial infarction, and aortic aneurysm. This situation is in addition to increased risk of mortality or poor quality of life of the remaining half of the affected individuals. In terms of age and gender-related distribution, males are more affected than females as young adult [1, 2]. This gender disparity disappears during adulthood, with males and females becoming equally affected, before switching in the other direction in individuals older than 65 y.o., when females become more affected than males [1, 2]. The reasons for this gender-related change in incidence with age are not completely understood, with loss of estrogen-based cardiovascular protection in females among the proposed hypotheses [1].

The etiology of hypertension is also complex spanning from endocrinopathies with abnormally elevated levels of one of the various pressure-controlling hormones (e.g., catecholamine, renin-angiotensin II-aldosterone axis, cortisol, and growth hormone) [3], to renal pathologies (e.g., Bartter's syndrome and Gitelman's syndrome), to primary cardiac hypertrophy, to conditions in which a clear cause-effect relationship cannot be establish (idiopathic hypertension). Accordingly, the pharmacological treatment of hypertension is multifaceted and based primarily on decreasing the circulating volume through the use of diuretics (e.g., furosemide, hydrochloro-thiazide, or amiloride) that inhibit different Na^+^ reabsorbing mechanisms in different portions of the nephron. Often, diuretic treatment is used in conjunction with angiotensin-converting-enzyme inhibitors (ACE inhibitors), Ca^2+^ channel blockers, or beta-blockers to regulate cardiac output, rate, and contraction and *α*1-adrenergic receptor blockers to control the tone of peripheral resistances, to surgical treatment to remove hormone hypersecreting adenomas, and to better address the underlying mechanism(s) responsible for the sustained increased in blood pressure [3, 4].

The complexity of hypertensive treatment and patient's compliance and responsiveness, however, result in significant costs for the health care system, without the guarantee of an effective medical control of the pathology. Consequently, the need for effective treatments that are economically more affordable and easy to comply with has increased progressively with the raising incidence of hypertension. Interest has shifted towards the implementation of diets that, while being affordable and easy to follow, do provide appropriate levels of macro- and micronutrients, and natural products (e.g., flavonoids) that can help in better controlling blood pressure and associated damaging processes such as oxidative stress.

In this regard, experimental and clinical evidence suggests that an appropriate intake of dietary magnesium can be beneficial in controlling blood pressure. Conceptually, intake of magnesium through the diet or as a supplement has the advantage of being a natural and required element for our cellular composition, of having virtual absence of side-effects, and being financially more affordable that other therapeutic agents, especially in developing countries. Practically, however, the utilization of magnesium in the contest of hypertension is still plagued by limited understanding of its true beneficial effects, limited bioavailability of some magnesium supplements, and the general misconception that because magnesium is abundantly present in our body and readily available through diet, the occurrence of magnesium deficiency is a rather rare condition.

In line with the nonpharmacological intent of this monographic issue, the present review will attempt to provide the necessary framework to understand the relevance of physiological magnesium levels for whole body well-being and for a better regulation of blood pressure.

## 2. Magnesium: Cellular Homeostasis and Transport

Magnesium is the fourth most abundant cation in the human body and the second most abundant intracellular cation after potassium [5]. Within the cell, magnesium ions (Mg^2+^) are highly compartmentalized within nucleus, mitochondria, endoplasmic and sarcoplasmic reticulum, cytoplasm, and possibly other organelles, although the assessment in the latter has been hampered by technical limitations [5]. Total Mg^2+^ concentrations in excess of 16-18 mM have been measured by a variety of approaches in each of the mentioned compartments [6], with the exception of cytoplasm where 4-5 mM Mg^2+^ is present in the form of a complex with ATP and other phosphonucleotides and between 0.8 and 1 mM is in the free form [6]. Magnesium ions regulate a broad variety of physiological functions, including Ca^2+^ channels, metabolomic receptors, ATPases, cell cycle, MAPKs, IP_3_ receptor, insulin signaling, and glucose transport and utilization, just to mention a few [5, 6]. At the cellular level and in the whole body, physiological Mg^2+^ contents are regulated through a series of extrusion and entry mechanisms, the operation of which is tightly controlled by a variety of hormones [5, 6]. Extrusion is mostly accomplished through a Na^+^/Mg^2+^ exchanger (Slc41A1) activated via cAMP-dependent-phosphorylation, which electrogenically transports 1Na^+^_in_:1Mg^2+^_out_ [5, 6]. Catecholamine, glucagon, and other cAMP-elevating hormones all promote Mg^2+^ extrusion in exchange for Na^+^ [7]. Supporting the importance of the Na^+^ electrochemical gradient in favoring Mg^2+^ extrusion, amiloride and other inhibitors of Na^+^ transport all inhibit Mg^2+^ extrusion through this exchanger [5–7]. Under conditions in which Na^+^ transport is inhibited, an alternative, less efficient, Na^+^-independent Mg^2+^ extrusion pathway becomes operative. This pathway is less well characterized, and not much is known about its activation, implying the possibility that different cells utilize different Na^+^-independent paths to maintain a basal level of Mg^2+^ extrusion [6, 7]. Entry of Mg^2+^ into the cell is accomplished through the operation of two distinct channels, termed TRPM6 and TRPM7 [5, 6]. While TRMP7 is ubiquitously expressed in eukaryotic cells, TRPM6 is more highly expressed in the distal convolute tubule of the nephron and in the descending colon, i.e., in locations where it can better control renal reabsorption and intestinal absorption to maintain whole body Mg^2+^ levels [5, 6]. Both these channels are maintained in the closed state by the binding of RACK1 at a specific site of the C-terminus [6]. Hormones or agents that activate protein kinase C within the cell can then scavenger RACK1 away from the channels, activating them [6]. Adding to the complexity of these channels, both TRPM6 and TRPM7 possess a *α*-kinase at their C-terminus, which becomes operative following Mg^2+^ entry and phosphorylates Ser and Thre residues in an alpha-helix loop [6]. Currently, only a handful of targets phosphorylated by these kinases have been identified [5, 6],* de facto* limiting our understanding of the role these kinases play in terms of whole cell function.

Because ~99% of Mg^2+^ is localized intracellularly in bones in soft tissue and ~1% is present in the circulation in both free (approximately 2/3) and bound (~1/3) form, changes in circulating Mg^2+^ levels cannot properly reflect the cellular status of the cation. Consequently, Mg^2+^ insufficiency or deficiency cannot be established using serum Mg^2+^ levels alone [5, 8], but assessment of urinary and intracellular Mg^2+^ levels should be included. Mg^2+^ deficiency, in fact, can be retrospectively identified based on the occurrence of an inverse relationship between the amount of Mg^2+^ eliminated through the urine and the amount of Mg^2+^ intake, and the variation in cellular Mg^2+^ content in more accessible pools such as red blood cells [8].

### 2.1. Dietary Mg^2+^ Levels and Intake

At the dietary level, Mg^2+^ is present in elevated content in green-leaf vegetables, whole grains, white potatoes, nuts, and legumes [9]. The current RDA (recommended dietary allowance) for magnesium ranges from 240 to 420 mg/day for males 31 to 70 y.o. and ~320 mg/d for females of comparable age range [10]. However, evidence is there indicating that ~60% of the US population does not consume the RDA for magnesium [11] and it is therefore considered to be magnesium insufficient if not frankly deficient. This assessment is supported by similar reports out of UK, Australia, Canada, and other industrialized countries [5], and the observation that dietary Mg^2+^ intake has declined by at least ~40% in the last 4 decades due to changes in food harvesting and processing, water purification, and overall dietary habits [12]. As a dietary supplement, magnesium is most commonly found as MgO (magnesium oxide). Other forms of mineral salts of Mg^2+^ supplements include Mg(OH)_2_ (magnesium hydroxide), magnesium bicarbonate, carbonate, phosphate, and sulfate. Alternatively, Mg^2+^ can be provided as an organic salt (bound to aspartate, citrate, gluconate, ascorbate, etc.) or chelated with amino acids [12]. While produced at a very low cost, several of the mineral salts of Mg^2+^ have a poor bioavailability (e.g., as low as 4% for magnesium oxide) [12], which greatly limit their effectiveness.

A certain level of magnesium deficiency has been reported in several chronic diseases including hypertension, diabetes, and metabolic syndrome [5]. Yet, it is undetermined whether magnesium deficiency precedes and perhaps contributes to the onset of the disease or is instead the result of the pathology. Irrespective of the time of appearance, however, due to its involvement in a broad range of cellular and physiological functions, magnesium insufficiency or deficiency can impair to a varying extent several of these functions and aggravates the progression of the pathology and its complications.

## 3. Magnesium and Hypertension

Several lines of evidence suggest a role of Mg^2+^ in inversely regulating blood pressure. Studies carried out in* in vitro* and* in vivo* models in the late 1940s or early 1950s first suggested a role of circulating Mg^2+^ in inhibiting catecholamine release from both peripheral and adrenal sources [13, 14]. Later studies validated these results and showed that infusion of Mg^2+^ significantly reduces the catecholamine-induced increases in systemic vascular resistance, systolic blood pressure, and diastolic blood pressure in a dose-dependent manner and increases coronary blood flow [reviewed in [15]].

A modulating effect of Mg^2+^ on vascular tone and reactivity, and consequently on blood pressure, is in line with the clinical observation that Mg^2+^ infusion decreases peripheral vascular resistance and blood pressure and can induce hypotension through vasodilatation [16, 17]. Increasing extracellular Mg^2+^ concentration promotes vasodilation, thereby increasing blood flow systemically and attenuates agonist-induced vasoconstriction [18, 19]. Conversely, decreasing extracellular Mg^2+^ concentration increases vascular tone and agonist-induced vasoconstriction [20, 21]. The exact mechanism behind these effects is not completely clear. Because Mg^2+^ acts as a natural Ca^2+^-channel blocker [15], it is conceivable that physiological extracellular Mg^2+^ concentrations would limit Ca^2+^ entry through the cell membrane and the increase in intracellular [Ca^2+^]i necessary for smooth muscle cells contraction and endocrine-regulated increase in vascular tone. Alternatively, owing to the involvement of extra- and intracellular Mg^2+^ in modulating a variety of signaling pathways [5–8], it is possible that an increase and a decrease in cellular Mg^2+^ concentrations can have major opposite implications for smooth muscle cells contraction and relaxation, whereby influencing vascular tone and blood pressure.

The effects of Mg^2+^ are not limited to vascular tone but influence vascular endothelial functions as well [22]. The vascular endothelium regulates vessel tone by releasing nitric oxide, endothelin-1, and prostacyclin PGI_2_ [22]. Magnesium ions have been reported to stimulate directly prostacyclin and nitric oxide production [23–25]. In addition, an inverse relationship between the levels of Mg^2+^ and endothelin-1 has been reported [26], further emphasizing the ability of magnesium to modulate vasodilatation through endothelium-dependent and independent mechanisms. A third possible mechanism whereby Mg^2+^ can modulate vascular tone and blood pressure is through its antioxidant and anti-inflammatory effects [27, 28]. Production of reactive oxygen species (ROS) and oxidative stress can be elevated in vascular smooth muscle cells, increasing vasoconstriction [29]. Conversely, the presence of physiological concentrations of Mg^2+^ would reduce ROS formation and antagonize the vasoconstriction effect. How exactly Mg^2+^ exerts this effect is not fully elucidated. Recently, Kolisek and collaborators [30] have hypothesized that the inverse relationship between ROS and Mg^2+^ levels is regulated through PARK7/DJ-1. This protein has antioxidant properties and tightly regulates cellular redox homeostasis. In addition, through androgen receptor activation, it would modulate the expression of SLC41A1, the main exchange mechanism responsible for Mg^2+^ extrusion in mammalian cells (see section on Magnesium: Cellular Homeostasis and Transport).

### 3.1. Is There a Role for Mg^2+^ in Controlling Hypertension in Human Patients?

Epidemiologic studies indicate that the consumption of “hard water” with high levels of magnesium is cardioprotective whereas consumption of “soft water” (i.e., water low in minerals including magnesium) is associated with hypertension and overall higher incidence of cardiovascular diseases. This inverse association was first observed by Joffres et al. in their Honolulu Heart study [31] and subsequently confirmed by other clinical studies [32, 33]. In particular, inverse relationship between magnesium levels and systolic and diastolic pressure values [32] and risk of death from hypertension [33] have been reported, in line with a similar inverse relationship between Mg^2+^ levels and circulating renin concentrations [34] and stiffened blood vessels [35].

The presence of an inverse relationship between circulating Mg^2+^ levels and the detected concentrations of endothelin-1, PGI_2_, ROS, NO, or renin, or systolic and diastolic blood pressure values, however, leaves unanswered the question as to whether magnesium supplementation can be therapeutic in restoring physiological and age-appropriate pressure values. In other words, are magnesium levels prophylactic, or they can also be therapeutic? The attempts to address this question have resulted in contradicting results, and only more recent studies appear to confirm beneficial therapeutic responses following administration of Mg^2+^ salts. The meta-analysis conducted by Dickinson and collaborators [36] assessed retrospectively 12 randomized controlled trials and indicates that diastolic pressure but not systolic pressure decreased to a significant extent as a result of magnesium supplementation [36]. Because of the limited quality of the trials included in the meta-analysis, however, the conclusion of the authors was that a causal association between magnesium supplementation and the decrease in blood pressure was weak, due to inherent bias [36]. More recently, the group of Kass has conducted a similar meta-analysis on more current studies. Twenty-two (22) trials with 23 sets of data were used, for a total of 1173 patients. While the majority of the studies assessed by Dickinson's group lasted for about 8 weeks, the studies analyzed by the group of Kass lasted anywhere from 3 to 24 weeks, with doses of Mg^2+^ ranging from 120 to ~970 mg/d [37]. The conclusion of this study was that higher doses of magnesium produced greater reduction in blood pressure, both systolic and diastolic values [37]. Similar conclusions have been attained more recently by Zhang and collaborators [38]. For their meta-analysis, this latter group assessed 34 trials involving more than 2000 participants, and magnesium supplementation (including a broad array of mineral and organic salts) ranged from 240 to 960 mg/d [38].

Based on these more recent studies [37, 38], it would appear that magnesium supplementation does achieve a statistical significant reduction in blood pressure, both systolic and diastolic values. It has to be noted that the reduction, while significant, ranges between 2 and 4 mmHg for either parameter. In clinical terms, it could be argued that such a reduction is rather small when compared to that attainable with other pharmacological treatments. Also, the presence of some variability in pressure reduction might imply that specific subgroups of patients are more (or less) magnesium-sensitive and therefore more prone to the beneficial effects of magnesium supplementation [37, 38]. At a first glance, it would appear that subgroups with higher magnesium sensitivity include patients of African descent, obese patients, patients with metabolic syndrome (see below) or preeclampsia (see also below), and patients with severe or malignant forms of hypertension [34–38]. Because our understanding of the mechanisms involved in controlling magnesium homeostasis and transport at the level of the cell and the whole body is still incomplete, it cannot be excluded that the “genetics” of magnesium homeostasis, alongside with diet composition, can be major factors in explaining this sensitivity and the effectiveness of magnesium supplementation.

### 3.2. Magnesium and Stroke

Cerebral insults (strokes) represent one of the most common, and feared, complications of hypertension, as they can result in paralysis, inability to speak, and difficulty in swallowing (with associated malnutrition) when they do not cause the immediate death of the patients. Strokes result from severe, not-controlled hypertension, affect ~800,000 new patients every year, and are responsible for ~140,000 deaths/year in the US alone [39]. Because of the association of this pathology with hypertension and the potential beneficial effect of magnesium supplementation in controlling hypertension, attention has been paid to the possibility that magnesium supplementation can indeed attenuate the incidence of stroke and/or their outcome. A meta-analysis study conducted by Larsson et al. [40] identified 7 prospective studies that could be used based on their criteria out of 163 articles screened. While the number of studies incorporated in the meta-analysis could be considered relatively small when compared to the starting number of articles screened, it did account for almost 6500 cases of stroke in a pool of participants of more than 240,000. The conclusion of the meta-analysis indicates once again the presence of a statistically significant inverse association between magnesium intake and risk of stroke. According to the study, an increment in magnesium intake of 100 mg/d correlates with an 8% reduction in the risk of stroke. Interestingly, this correlation applies only to ischemic strokes and not to hemorrhagic strokes [40]. While a causal relationship could not be fully validated, it is worth noting that Han and collaborators have recently reported a 5% reduction in the risk of hypertension for a similar (100mg/d) increment in magnesium intake [41].

## 4. Magnesium and Metabolic Syndrome

The incidence of metabolic syndrome in the human population shows a trend similar to that of hypertension, affecting more than 500 million people worldwide [42]. Haller was the first to introduce the term in 1977 to describe a pathological condition characterized by the association of obesity, diabetes mellitus, hyperlipoproteinemia, hyperuricemia, and hepatic steatosis [43]. Currently, the pathology is diagnosed based on the presence of at least 3 of the following 5 criteria: (1) central obesity (waist circumference ≥ 102 cm or 40 inches for males, and ≥ 88 cm or 35 inches for females); (2) hypertriglyceridemia (TG ≥ 1.7 mmol/L or 150 mg/dl); (3) dyslipidemia (HDL-C < 40 mg/dL for males, and < 50 mg/dL for females) with slightly or markedly elevated total cholesterol and LDL; (4) blood pressure ≥ 130/85 mmHg (or treated for hypertension); and (5) fasting plasma glucose ≥ 6.1 mmol/L (110 mg/dl) [44].

As mentioned above, an increase in systolic and diastolic blood pressure values represents one of the diagnostic criteria for this condition. Hypomagnesemia [45] and intracellular magnesium deficiency [46] are also common features of the pathology. The cause(s) for the onset of metabolic syndrome are not elucidated: for a period of time, the condition was termed Syndrome X to highlight its obscure etiology. Because of the altered triglyceride and cholesterol profiles and the presence of slightly elevated glycemia, the metabolic syndrome is currently considered the result of incipient insulin resistance [44], and progression towards type-2 diabetes mellitus is a typical complication of the syndrome [47]. Because insulin favors Mg^2+^ accumulation within cells [5, 6], reduced cellular Mg^2+^ levels in the context of insulin resistance are to be expected. According to the CARDIA study, a magnesium enriched diet or magnesium supplementation appear effective in reducing the risk of metabolic syndrome and its progression towards diabetes and cardiovascular complications [48].

## 5. Magnesium and Preeclampsia

The term preeclampsia refers to a clinical disorder during pregnancy characterized by hypertension after 20 weeks of gestation, with marked proteinuria [49]. The disorder affects about 5% of delivery and when left untreated it develops into eclampsia, with occurrence of tonic-clonic seizures for about 1 min, followed by a period of confusion or coma [49, 50]. Major complications of eclampsia are as follows: aspiration pneumonia, cerebral hemorrhage, kidney failure, and cardiac arrest [50]. Eclampsia affects about 1.5% or all the deliveries, with a mortality rate around 1% of all the affected women [50]. In 2015, the last year for which we have reliable collected data, eclampsia accounted to ~47,000 deaths in the world [51].

Magnesium sulfate constitutes the treatment of choice for the prevention of preeclampsia related convulsions. The first report of its effectiveness was by Pritchard in 1955 [52]. As its anticonvulsion use became more common and accepted [53], administration of magnesium sulfate has been observed to result in better outcome than other anticonvulsive (e.g., diazepam) [54]. Anticonvulsive drugs such as diazepam or phenytoin [53, 54] are still used for the treatment of eclampsia as coadjuvants of magnesium sulfate treatment. The addition of these therapeutic agents helps in maintaining magnesium dosage and circulating magnesium levels in an optimal therapeutic range, thus preventing magnesium-related toxic side-effects such as paralysis of maternal thoracic muscles and respiratory depression, which could occur at high doses of magnesium sulfate if this was the only therapeutic agent used [53]

Treatment of preeclampsia with magnesium sulfate has been reported to improve endothelial function. This improvement can be due to the previously described beneficial effects of magnesium on vascular tone (e.g., reduced stiffness and reduced Ca^2+^ entry), vascular responsiveness to endothelin-1, renin, and ROS production, and overall vascular function, including increased nitric oxide and PGI_2_ production, which promote vasodilation and inhibit platelet aggregation, respectively [23–26]. Yet, it still remains controversial whether women undergoing preeclampsia have lower than normal circulating levels of Mg^2+^ and therefore are more exposed to the vascular irregularities and hypertone prevented by Mg^2+^. Some studies have indeed reported decreased serum and intracellular Mg^2+^ levels [55, 56] whereas other studies have failed to identify similar differences between preeclamptic and healthy gravidas [57, 58]. Also in this clinical scenario, the reason(s) for such a discrepancy is/are not apparent, although patients' heterogeneity and perhaps dietary intake are possible confounders.

Despite the inconsistency in basal serum and cellular Mg^2+^ levels between healthy women and women with preeclampsia, observation by Standley and collaborators suggests that magnesium levels can still be used as a predictive/prognostic tool [59]. In studying magnesium levels at different gestational ages, this group observed that the levels of the cation decrease in both preeclamptic and healthy pregnancies, but the decrease in preeclamptic women occurs at an earlier stage as compared to the healthy counterparts and could therefore be utilized as a marker of severity of the pathology [59].

## 6. Conclusions and Perspectives

In this review, we have attempted to provide a general understanding of how human cells control cellular and circulating magnesium levels, the importance of these levels for wellness in general and blood pressure levels in particular, and how diet and dietary supplements can be utilized effectively to maintain physiological levels of magnesium. We have also attempted to provide an appreciation of the complexity surrounding the prophylactic and possibly therapeutic use of magnesium supplementation in hypertension, and hypertension-associated pathologies such as stroke, metabolic syndrome, and preeclampsia/eclampsia.

Despite the inherent difficulty to provide a clear cut approach and validity to the use of magnesium supplementation as a therapeutic tool, it is apparent from our reviewing of the mentioned pathologies that magnesium supplementation does have a role as a coadjuvant of more established pharmacological tools currently utilized in the various fields. The low cost of production and the virtual absence of side-effects in the normal range of doses more commonly utilized (e.g., up to 960 mg/day) add to the “appeal” of a routine use of this mineral as a dietary supplement, especially in areas where health care costs, availability of more expensive drugs, and ultimately compliance of the patients to a given therapeutic protocol represent critical factors hampering the effectiveness of medical intervention.

## References

[1] http://www.who.int/topics/hypertension/en/, May 12, 2018

[2] Merai R., Siegel C., Rakotz M. (2016). CDC grand rounds: a public health approach to detect and control hypertension. *Morbidity and Mortality Weekly Report (MMWR)*.

[3] https://www.froedtert.com/endocrinology/endocrine-hypertension, May 12, 2018

[4] Whelton P. K., Carey R. M., Aronow W. S. (2018). 2017 ACC/AHA/AAPA/ABC/ACPM/AGS/APhA/ASH/ASPC/NMA/PCNA guideline for the prevention, detection, evaluation, and management of high blood pressure in adults. *Journal of the American College of Cardiology*.

[5] Romani A. M. P. (2013). Magnesium in health and disease. *Metal Ions in Life Sciences*.

[6] Romani A., Scarpa A. (1992). Regulation of cell magnesium. *Archives of Biochemistry and Biophysics*.

[7] Scarpa A. (2000). Regulation of cellular magnesium. *Frontiers in Bioscience*.

[8] Costello R. B., Elin R. J., Rosanoff A. (2016). Perspective: the case for an evidence-based reference interval for serum magnesium: the time has come. *Advances in Nutrition*.

[9] Volpe S. L. (2013). Magnesium in disease prevention and overall health. *Advances in Nutrition*.

[10] Ross A. C., Manson J. E., Abrams S. A. (2011). The 2011 report on dietary reference intakes for calcium and vitamin D from the institute of medicine: what clinicians need to know. *The Journal of Clinical Endocrinology & Metabolism*.

[11] Nielsen F. H. (2010). Magnesium, inflammation, and obesity in chronic disease. *Nutrition Reviews*.

[12] https://ods.od.nih.gov/factsheets/Magnesium-HealthProfessional/, May 12, 2018

[13] Hutter O. F., Kostial K. (1954). Effect of magnesium and calcium ions on the release of acetylcholine. *The Journal of Physiology*.

[14] Standbury J. B. (1948). The blocking action of magnesium on sympathetic ganglia. *Journal of Pharmacology and Experimental Therapeutics*.

[15] Dubé L., Granry JC. (2003). The therapeutic use of magnesium in anesthesiology, intensive care and emergency medicine: a review. *Canadian Journal of Anesthesia*.

[16] Akhtar M. I., Ullah H., Hamid M. (2011). Magnesium a drug of diverse use. *Journal of Pakistan Medical Association*.

[17] Resnick L. M., Gupta R. K., DiFabio B. (1994). Intracellular ionic consequences of dietary salt loading in essential hypertension. Relation to blood pressure and effects of calcium channel blockade. *The Journal of Clinical Investigation*.

[18] Tammaro P., Smith A. L., Crowley B. L., Smirnov S. V. (2005). Modulation of the voltage-dependent K + current by intracellular Mg 2+ in rat aortic smooth muscle cells. *Cardiovascular Research*.

[19] Touyz R. M., Yao G. (2003). Modulation of vascular smooth muscle cell growth by magnesium-role of mitogen-activated protein kinases. *Journal of Cellular Physiology*.

[20] Laurant P., Touyz R. M., Schiffrin E. L. (1997). Effect of magnesium on vascular tone and reactivity in pressurized mesenteric resistance arteries from spontaneously hypertensive rats. *Canadian Journal of Physiology and Pharmacology*.

[21] Yoshimura M., Oshima T., Matsuura H., Ishida T., Kambe M., Kajiyama G. (1997). Extracellular Mg2+ inhibits capacitative Ca2+ entry in vascular smooth muscle cells. *Circulation*.

[22] Maier J. A., Bernardini D., Rayssiguier Y., Mazur A. (2004). High concentrations of magnesium modulate vascular endothelial cell behaviour in vitro. *Biochimica et Biophysica Acta (BBA)—Molecular Basis of Disease*.

[23] Satake K., Lee J.-D., Shimizu H. (2004). Effects of magnesium on prostacyclin synthesis and intracellular free calcium concentration in vascular cells. *Magnesium Research*.

[24] Soltani N., Keshavarz M., Sohanaki H., Asl S. Z., Dehpour A. R. (2005). Relaxatory effect of magnesium on mesenteric vascular beds differs from normal and streptozotocin induced diabetic rats. *European Journal of Pharmacology*.

[25] Landau R., Scott J. A., Smiley R. M. (2004). Magnesium-induced vasodilation in the dorsal hand vein. *BJOG: An International Journal of Obstetrics & Gynaecology*.

[26] Laurant P., Berthelot A. (1996). Endothelin-1-induced contraction in isolated aortae from normotensive and DOCA-salt hypertensive rats: effect of magnesium. *British Journal of Pharmacology*.

[27] Weglicki W. B., Phillips T. M., Freedman A. M., Cassidy M. M., Dickens B. F. (1992). Magnesium-deficiency elevates circulating levels of inflammatory cytokines and endothelin. *Molecular and Cellular Biochemistry*.

[28] Weglicki W. B., Mak I. T., Kramer J. H. (1996). Role of free radicals and substance P in magnesium deficiency. *Cardiovascular Research*.

[29] Taniyama Y., Griendling K. K. (2003). Reactive oxygen species in the vasculature: molecular and cellular mechanisms. *Hypertension*.

[30] Kolisek M., Montezano A. C., Sponder G. (2015). PARK7/DJ-1 dysregulation by oxidative stress leads to magnesium deficiency: implications in degenerative and chronic diseases. *Clinical Science*.

[31] Joffres M. R., Reed D. M., Yano K. (1987). Relationship of magnesium intake and other dietary factors to blood pressure: the Honolulu heart study. *American Journal of Clinical Nutrition*.

[32] Kao W. H. L., Folsom A. R., Nieto F. J., Mo J.-P., Watson R. L., Brancati F. L. (1999). Serum and dietary magnesium and the risk for type 2 diabetes mellitus: the atherosclerosis risk in communities study. *JAMA Internal Medicine*.

[33] Yang C.-Y., Chiu H.-F. (1999). Calcium and magnesium in drinking water and the risk of death from hypertension. *American Journal of Hypertension*.

[34] Resnick L. M., Laragh J. H., Sealey J. E., Alderman M. H. (1983). Divalent cations in essential hypertension: relations between serum ionized calcium, magnesium, and plasma renin activity. *The New England Journal of Medicine*.

[35] Resnick L. M., Militianu D., Cunnings A. J., Pipe J. G., Evelhoch J. L., Soulen R. L. (1997). Direct magnetic resonance determination of aortic distensibility in essential hypertension: relation to age, abdominal visceral fat, and in situ intracellular free magnesium. *Hypertension*.

[36] Dickinson H. O., Nicolson D. J., Campbell F. (2006). Magnesium supplements for the management of essential hypertension in adults. *Cochrane Database of Systematic Reviews*.

[37] Kass L., Weekes J., Carpenter L. (2012). Effect of magnesium supplementation on blood pressure: a meta-analysis. *European Journal of Clinical Nutrition*.

[38] Zhang X., Li Y., Del Gobbo L. C. (2016). Effects of magnesium supplementation on blood pressure: a meta-analysis of randomized double-blind placebo-controlled trials. *Hypertension*.

[39] https://www.cdc.gov/media/releases/2017/p0906-vs-stroke.html, January 2018

[40] Larsson S. C., Orsini N., Wolk A. (2012). Dietary magnesium intake and risk of stroke: a meta-analysis of prospective studies. *American Journal of Clinical Nutrition*.

[41] Han H., Fang X., Wei X. (2017). Dose-response relationship between dietary magnesium intake, serum magnesium concentration and risk of hypertension: a systematic review and meta-analysis of prospective cohort studies. *Nutrition Journal*.

[42] http://www.cdc.gov/obesity/data/adult.html, January 2018

[43] Haller H. (1977). Epidermiology and associated risk factors of hyperlipoproteinemia. *Zeitschrift für die gesamte innere Medizin und ihre Grenzgebiete*.

[44] Grundy S. M., Brewer H. B., Cleeman J. I., Smith S. C., Lenfant C. (2004). Definition of metabolic syndrome report of the national heart, lung, and blood institute/american heart association conference on scientific issues related to definition. *Circulation*.

[45] Lopez-Ridaura R., Willett W. C., Rimm E. B. (2004). Magnesium intake and risk of type 2 diabetes in men and women. *Diabetes Care*.

[46] Guerrero-Romero F., Rodríguez-Morán M. (2002). Low serum magnesium levels and metabolic syndrome. *Acta Diabetologica*.

[47] Reaven G. M. (2005). The insulin resistance syndrome: definition and dietary approaches to treatment. *Annual Review of Nutrition*.

[48] He K., Liu K., Daviglus M. L. (2006). Magnesium intake and incidence of metabolic syndrome among young adults. *Circulation*.

[49] Lambert G., Brichant J. F., Hartstein G., Bonhomme V., Dewandre P. Y. (2014). Preeclampsia: an update. *Acta Anaesthesiologica Belgica*.

[50] Williams (2014). *Obstetrics*.

[51] GBD 2015 Mortality and Causes of Death Collaborators (2015). Global, regional, and national life expectancy, all-cause mortality, and cause-specific mortality for 249 causes of death, 1980-2015: a systematic analysis for the Global Burden of Disease Study. *Lancet*.

[52] Pritchard J. A. (1955). The use of the magnesium ion in the management of eclamptogenic toxemias. *Surgery, Gynecology & Obstetrics—Journals*.

[53] Smith J. M., Lowe R. F., Fullerton J., Currie S. M., Harris L., Felker-Kantor E. (2013). An integrative review of the side effects related to the use of magnesium sulfate for pre-eclampsia and eclampsia management. *BMC Pregnancy and Childbirth*.

[54] Duley L., Henderson-Smart D. J., Walker G. J., Chou D. (2010). Magnesium sulphate versus diazepam for eclampsia. *Cochrane Database of Systematic Reviews (Online)*.

[55] Seydoux J., Girardin E., Paunier L., Béguin F. (1992). Serum and intracellular magnesium during normal pregnancy and in patients with pre‐eclampsia. *BJOG: An International Journal of Obstetrics & Gynaecology*.

[56] He L., Lang L., Li Y., Liu Q., Yao Y. (2016). Comparison of serum zinc, calcium, and magnesium concentrations in women with pregnancy-induced hypertension and healthy pregnant women: a meta-analysis. *Hypertension in Pregnancy*.

[57] Sanders R., Konijnenberg A., Huijgen H. J., Wolf H., Boer K., Sanders G. T. (1999). Intracellular and extracellular, ionized and total magnesium in pre-eclampsia and uncomplicated pregnancy. *Clinical Chemistry and Laboratory Medicine*.

[58] Frenkel Y., Weiss M., Shefi M., Lusky A., Mashiach S., Dolev E. (1994). Mononuclear cell magnesium content remains unchanged in various hypertensive disorders of pregnancy. *Gynecologic and Obstetric Investigation*.

[59] Standley C. A., Whitty J. E., Mason B. A., Cotton D. B. (1997). Serum ionized magnesium levels in normal and preeclamptic gestation. *Obstetrics & Gynecology*.

